# Revealing the Intrinsic Electronic Structure of 3D Half‐Heusler Thermoelectric Materials by Angle‐Resolved Photoemission Spectroscopy

**DOI:** 10.1002/advs.201902409

**Published:** 2019-11-06

**Authors:** Chenguang Fu, Mengyu Yao, Xi Chen, Lucky Zaehir Maulana, Xin Li, Jiong Yang, Kazuki Imasato, Fengfeng Zhu, Guowei Li, Gudrun Auffermann, Ulrich Burkhardt, Walter Schnelle, Jianshi Zhou, Tiejun Zhu, Xinbing Zhao, Ming Shi, Martin Dressel, Artem V. Pronin, G. Jeffrey Snyder, Claudia Felser

**Affiliations:** ^1^ Max Planck Institute for Chemical Physics of Solids Nöthnitzer Str. 40 01187 Dresden Germany; ^2^ Swiss Light Source Paul Scherrer Institut CH‐5232 Villigen Switzerland; ^3^ Materials Science and Engineering Program Texas Materials Institute The University of Texas at Austin Austin TX 78712 USA; ^4^ 1. Physikalisches Institut Universität Stuttgart 70569 Stuttgart Germany; ^5^ Materials Genome Institute Shanghai University 99 Shangda Road Shanghai 200444 China; ^6^ Department of Materials Science and Engineering Northwestern University Evanston IL 60208 USA; ^7^ Jülich Center for Neutron Science JCNS at Heinz Maier‐Leibnitz Zentrum (MLZ) Forschungszentrum Jülich GmbH 85747 Garching Germany; ^8^ Department of Physics and Astronomy Shanghai Jiao Tong University Shanghai 200240 China; ^9^ State Key Laboratory of Silicon Materials and School of Materials Science and Engineering Zhejiang University Hangzhou 310027 China; ^10^ Center for Nanoscale Systems (CNS) Faculty of Arts and Sciences Harvard University Cambridge MA 02138 USA

**Keywords:** bandgap, electronic structure, half‐Heusler compounds, thermoelectric properties

## Abstract

Accurate determination of the intrinsic electronic structure of thermoelectric materials is a prerequisite for utilizing an electronic band engineering strategy to improve their thermoelectric performance. Herein, with high‐resolution angle‐resolved photoemission spectroscopy (ARPES), the intrinsic electronic structure of the 3D half‐Heusler thermoelectric material ZrNiSn is revealed. An unexpectedly large intrinsic bandgap is directly observed by ARPES and is further confirmed by electrical and optical measurements and first‐principles calculations. Moreover, a large anisotropic conduction band with an anisotropic factor of 6 is identified by ARPES and attributed to be one of the most important reasons leading to the high thermoelectric performance of ZrNiSn. These successful findings rely on the grown high‐quality single crystals, which have fewer Ni interstitial defects and negligible in‐gap states on the electronic structure. This work demonstrates a realistic paradigm to investigate the electronic structure of 3D solid materials by using ARPES and provides new insights into the intrinsic electronic structure of the half‐Heusler system benefiting further optimization of thermoelectric performance.

## Graphical abstract

Herein, the intrinsic electronic structure of archetypical 3D ZrNiSn half‐Heusler compound is resolved using angle‐resolved photoemission spectroscopy. Unexpectedly large intrinsic bandgap and anisotropic conduction characteristics are directly revealed. These results provide new insights into the origin of high power factor of half‐Heuslers and offer a new strategy to further optimize their thermoelectric properties.

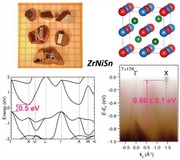

The knowledge of electronic structure is essential for understanding the physical properties of solids, such as the electrical resistivity ρ, thermopower α, and optical absorption, which lay the foundation of modern solid‐state electronic devices, such as solar cells and thermoelectric modules. It has been established since the middle of the 20th century that the best thermoelectric materials are narrow‐bandgap semiconductors due to their characteristic electronic structure.^1^ The bandgap, *E*_g_, a foremost parameter derived from the electronic structure, plays a vital role in determining the peak value of the figure of merit *zT* of a thermoelectric semiconductor.^2^, ^3^ With increasing temperature, the thermally excited minority carriers cross the bandgap, rapidly deteriorate the thermopower, and cause an increase of electrical and thermal conductivities,^3^ making the *zT* peaks at a certain temperature. Therefore, higher *E*_g_ generally leads to higher peak *zT* for a certain thermoelectric material. The band effective mass, *m*_b_*, another important parameter derived from the electronic structure, has an opposite contribution to carrier mobility and thermopower.^4^, ^5^ A probable solution to this dilemma is to take advantage of band anisotropy. The dispersive band guarantees high carrier mobility while the flat band direction serves as the carriers' reservoir securing good thermopower.^4^, ^6^ Therefore, accurate determination of the electronic structure is significant for understanding the high thermoelectric performance of good thermoelectric materials.^3^, ^6^, ^7^

Half‐Heusler compounds have recently attracted considerable attention from the thermoelectric community.^8^, ^9^ Three representative systems, i.e., *M*NiSn (*M* = Ti, Zr, and Hf),^10^, ^11^ *M*CoSb,^12^, ^13^ and *R*FeSb (*R* = V, Nb, and Ta),^14^, ^15^ have been developed as good thermoelectric materials with *zT* values above unity. These good results make the half‐Heusler system very promising for high‐temperature power generation especially with their good mechanical properties and thermal stability. Different from some other good thermoelectric materials, one remarkable feature contributing to the high *zT* of half‐Heusler compounds is their high electrical power factor (PF = α^2^/ρ). Therefore, in‐depth experimental investigation of the electronic structure and accurate acquisition of the related parameters (e.g., *E*_g_, *m*_b_*, and anisotropic factor) would be significant for understanding the good thermoelectric performance of half‐Heusler compounds. Particularly, under the context of that lattice thermal conductivity of thermoelectric materials has been well tamed through multiple strategies.^16^

*M*NiSn, as the first found half‐Heusler thermoelectric system,^17^ serves as a ripe platform for exploring the intrinsic origin of high PF. In recent years, large density of states near Fermi energy,^18^ low deformation potential,^19^ and high band degeneracy,^20^ have been recognized as important factors that contribute to high PF. However, there are still some unresolved problems related to the electronic structure of *M*NiSn, such as “the real *E*_g_ of *M*NiSn.” This problem can be dated back to the original work of Aliev et al.,^16^, ^17^ who first found that the intermetallic compounds *M*NiSn consisting of three metallic elements show a semiconducting behavior. Using the temperature‐dependent resistivity and optical transmittance and reflectance measurements, they reported an *E*_g_ of approximately 0.2 eV based on polycrystalline samples synthesized by arc‐melting, which was confirmed by subsequent studies.^21^ All these results are summarized in **Figure** **1**a. However, a dilemma emerged when the first‐principles calculations were carried out to study the electronic structure of ZrNiSn. In 1995, Öğüt and Rabe reported a calculated *E*_g_ of 0.5 eV,^22^ which was later validated by following theoretical calculations.^18^, ^23^ It is easily found that the experimental *E*_g_ is much smaller than the calculated one. This is highly unexpected because the first‐principles calculations usually underestimate, not overestimate the bandgap of a material.^24^, ^25^ With the considerable experimental results, it is found that in nominally stoichiometric polycrystalline ZrNiSn, generally synthesized by high‐temperature technique (e.g., arc melting, induction melting, and levitation melting), there is always some excess Ni occupying the interstitial sites.^25^, ^26^, ^27^, ^28^ As a result, the actual composition becomes ZrNi_1+_ *_x_*Sn (*x* is about 5%). These interstitial Ni atoms form additional in‐gap states in the forbidden gap,^29^ which lead to that the observable *E*_g_ is the gap between the conduction band (CB) and the in‐gap states, instead of the valence band (VB),^25^ as schematically shown in Figure 1b. Although this finding well explains the difference between the observable experimental *E*_g_ and the calculated one, the *real E_g_* of ZrNiSn, *the gap between CB and VB*, remains unresolvable. Furthermore, there is still no experimental work that directly investigates the CB of ZrNiSn using angle‐resolved photoemission spectroscopy (ARPES) technique, which is significant for understanding its high power factor and *zT* since ZrNiSn is a good *n*‐type thermoelectric material.

**Figure 1:**
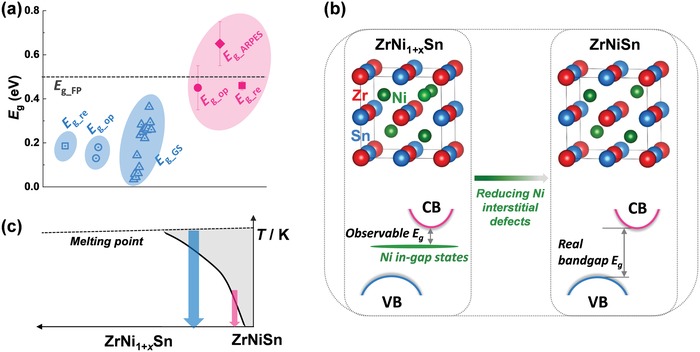
a) Bandgap *E*_g_ of ZrNiSn, identified by multiple methods. The blue regions show the *E*_g_ of polycrystalline samples from the previous studies.^17^, ^21^ The red region shows the *E*_g_ obtained from the single crystals in this work. *E*_g_re_ is obtained from the resistivity measurement: ρ ≈ exp(*E*_g_re_/2*k*_B_ *T*). *E*_g_op_ is derived from the optical measurement. *E*_g_GS_ is the Goldsmid–Sharp bandgap.^34^ *E*_g___ARPES_ is derived from the ARPES study. *E*_g_FP_ is from the first‐principles calculations. b) Crystal structure of ZrNi_1+_ *_x_*Sn (left panel) and ZrNiSn (right panel), respectively, drawn with VESTA.^35^ The corresponding electronic structure is exhibited below the crystal structure. CB and VB denote the conduction and valence bands, respectively. c) Schematic showing the pseudo‐binary phase diagram of ZrNi_1+_ *_x_*Sn and ZrNiSn.^33^ The blue and red arrows indicate high‐temperature and low‐temperature preparation techniques, respectively.

These above‐mentioned issues principally can be resolved by performing high‐resolution ARPES studies on ZrNiSn, which enable direct observation of the intrinsic electronic structure.^30^ Recently, ARPES experiments have been carried out to investigate the quasi‐1D and 2D thermoelectric materials CsBi_4_Te_6_ 31 and SnSe,^32^ respectively, and significantly different intrinsic electronic structure was revealed. However, it is more challenging to perform ARPES study on 3D crystals in contrast to the 2D layered compounds. A big technical problem is how to cleave the crystal to secure a well‐ordered surface. Furthermore, for the current study, to observe the CB of ZrNiSn, heavily doped *n*‐type single crystals are needed because only occupied electronic states below the Fermi level can be resolved by ARPES. Additionally, the existence of Ni in‐gap states in the forbidden gap might cause difficulties in distinguishing the CB and VB. In this work, we have overcome these challenges and first revealed the intrinsic electronic structure of 3D half‐Heusler compound ZrNiSn by ARPES. An unexpectedly large intrinsic bandgap and anisotropic CB are directly observed by ARPES.

Previous studies have demonstrated that the existence of Ni in‐gap states in ZrNi_1+_ *_x_*Sn makes the observable *E*_g_ much smaller than the real one,^25^ as shown in Figure 1b. Therefore, to observe the intrinsic *E*_g_ of ZrNiSn, it is crucial to growing the crystals with reduced Ni interstitial defects. From the pseudo‐binary phase diagram of the ZrNi_1+_ *_x_*Sn and ZrNiSn system (Figure 1c), it is found that there is an obvious increase in the solubility of excess Ni in ZrNi_1+_ *_x_*Sn with increasing temperature. As an intermetallic compound, ZrNiSn polycrystalline ingots were usually synthesized by using arc melting, induction melting, or levitation melting.^9^, ^10^, ^28^ The ingots are formed after rapid cooling from its liquid state (the melting point of ZrNiSn is 1465 °C),^33^ as indicated by the thick blue arrow in Figure 1c. Thus, more Ni interstitial defects might be easily formed in the obtained polycrystalline samples. To obtain the crystals with less Ni interstitial defects, it is crucial to growing the crystals at lower temperatures with a slow‐growing rate, as shown by the thin red arrow in Figure 1c.

Herein, undoped and Sb‐doped ZrNiSn single crystals have been grown with a low‐temperature preparation technique, i.e., solidification in a Sn‐rich melt by slow cooling (2 °C h^−1^) from 1100 to 650 °C, details can be found in the Experimental Section. The optical image of the as‐grown crystals, which have a typical length of 2 mm and shiny surfaces, is shown in **Figure** **2**a. Powder X‐ray diffraction (XRD) patterns of undoped ZrNiSn and Sb‐doped ZrNiSn_0.97_Sb_0.03_ crystals are shown in Figure S1 (Supporting Information) and no obvious impurity phase is observed. The crystallinity and orientation of the crystals are further checked with Laue diffraction measurement. The obtained Laue diffraction pattern can be indexed based on the $F\bar{4}3m$ space group and superposed well with a theoretically simulated pattern (Figure S2, Supporting Information). The optical and backscattered scanning electron microscope images of the polished ZrNiSn and ZrNiSn_0.97_Sb_0.03_ single crystals in Figures S3 and S4 (Supporting Information), respectively, show the homogenous phase. The lattice parameter of ZrNiSn single crystal is calculated to be 6.1033(2) Å at 300 K (Table S1, Supporting Information), which is appreciably smaller than the value of 6.1141(1) Å for ZrNi_1.046_Sn,^26^ indicating that the studied single crystals might have fewer Ni interstitial defects. The actual compositions of the undoped ZrNiSn and Sb‐doped ZrNiSn_0.97_Sb_0.03_ single crystals were carefully examined using energy‐dispersive X‐ray spectroscopy (EDX) and wavelength‐dispersive X‐ray spectroscopy (WDX), respectively (details are in Tables S2 and S3, Supporting Information). The results indicate a smaller amount of excess Ni (about 1–2%) in the studied single crystals, compared to that of about 5% in the polycrystalline crystals using high‐temperature preparation technique.^25^, ^26^ Moreover, we found the actual composition of the as‐grown single crystals still shows similar excess Ni of about 1–2% even under the condition that the initial Ni content is changed. For further validation, the inductively coupled plasma‐optical emission spectroscopy (ICP‐OES) analysis was carried out on the undoped single crystals. The actual composition is identified as Zr_0.995_Ni_1.009_Sn_0.996_ with a standard deviation of approximately 1% for each element (Table S4, Supporting Information), agreeing well with the WDX results. Therefore, the lattice parameter and compositional analyses demonstrate the high‐quality of the as‐grown single crystals with fewer Ni interstitial defects, paving a good foundation for carrying out the ARPES study.

**Figure 2:**
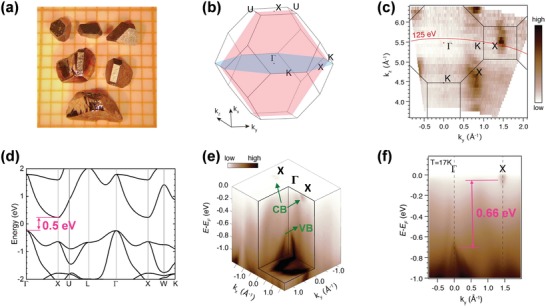
a) Optical image of as‐grown single crystals on a 1 × 1 mm^2^ grid. b) Brillouin zone with high‐symmetry points. In the momentum coordinate, *k_x_*–*k_y_* is set up within the red plane, while the *k_y_*–*k_z_* plane in blue. c) Fermi surface intensity plot in the *k_y_*–*k_z_* plane at *k_x_* = 0, acquired with linear horizontal photon with photon energy ranging from 60 to 160 eV. The black lines represent the Brillouin zone in the *k_y_*–*k_z_* plane. d) Calculated electronic band structure for ZrNiSn. e) 3D intensity plot of the photoemission data, showing the Fermi surface and electronic structure of ZrNiSn, including two electron pockets at *X* point and a hole pocket at *Γ* point. f) ARPES intensity plots along *Γ*–*X* direction, taken with the photon energy of 125 eV as indicated in (c).

For ARPES experiments, Sb‐doped *n*‐type ZrNiSn_0.97_Sb_0.03_ single crystals are chosen so that the CB could be observed. The crystals are first oriented along the [110] direction and then cut into bar sharp. To secure a well‐ordered cleavage surface of these 3D crystals, a small incision perpendicular to [110] direction is first cut using a 50 µm wire saw (Figure S5, Supporting Information). With this pre‐processing of the crystals, we have reached a high success rate to cleave this 3D crystal, as evidenced by the shiny and flat (110) cleavage surface (Figure S5b, Supporting Information). The ARPES measurements are thus carried out on the (110) surface, which corresponds to the red plane in the bulk Brillouin zone (Figure 2b). With the photon energy ranging from 60 to 160 eV, we can capture the information of the electronic structure of ZrNiSn in a whole Brillouin zone. The photon energy‐dependent Fermi surface map of ZrNiSn_0.97_Sb_0.03_ in *k_y_*–*k_z_* plane is presented in Figure 2c. The data show strong dispersion along *k_z_* direction, indicating the bulk electronic band information. The CB at *X* point is clearly resolved, agreeing with the calculated electronic structure (Figure 2d). The energy‐dependent 3D intensity plot of the photoemission data in *k_x_*–*k_y_* plane is presented in Figure 2d, which show clear CB and VB.

For further analysis of the intrinsic electronic structure, 2D ARPES intensity plot along *Γ*–*X* direction acquired with the photon energy of 125 eV at 17 K is exhibited in Figure 2f. This experimental electronic structure enables us to check whether the Ni in‐gap states exist or not. As previously reported by Zeier et al.,^25^ 5% excess Ni on interstitial defects are expected to produce flat in‐gap states within the *k_x_*–*k_y_* plane. However, such flat bands are not observed in the forbidden gap (Figure 2f), indicating a negligible effect of Ni in‐gap states in the studied single crystals. Without the obvious effect of Ni in‐gap states, the experimental electronic structure offers a chance to resolve the real *E*_g_ between CB minimum and VB maximum. To accurately characterize the *E*_g_, we consider the energy distribution curves at the *Γ* and *X* points (Figure S6, Supporting Information). The energy distribution curves show a peak at −0.71 eV, corresponding to the VB maximum at *Γ* point. The intensity peak of the electron pocket is at −0.05 eV, pointing to the CB minimum at *X* point. As a result, the *E*_g_‐ARPES of ZrNiSn is derived as 0.66 ± 0.1 eV. The error bar is set by the energy resolution of the ARPES experiment. This unexpectedly large *E*_g_ARPES_ is approximately two to three times higher than the values previously reported on the polycrystalline samples synthesized by high‐temperature technique (as shown in Figure 1a).

The experimental electronic structure from ARPES enables us to directly acquire the electronic structure‐related parameters of ZrNiSn, i.e., band effective mass and anisotropic factor, which are important for understanding the origin of high PF in half‐Heusler thermoelectric materials. The Fermi surface mapping of ZrNiSn_0.97_Sb_0.03_ in the *k_x_*–*k_y_* plane is presented in **Figure** **3**a. The CB at the *X* point shows an obvious anisotropy with an ellipsoid shape, which is much flatter along the *X*–*Γ* direction (*k_x_* axis) than that along the *X*–*U* direction (*k_y_* axis). To confirm this result, the Fermi surface was further calculated using the first‐principles calculations at a carrier concentration of 5 × 10^20^ cm^−3^, matching the experimental *n*_H_ of ZrNiSn_0.97_Sb_0.03_ (Figure S7, Supporting Information). Figure 3b shows the calculated electron pocket with the same Brillouin zone setup with Figure 3a, which also exhibits an ellipsoidal Fermi surface. To obtain the band effective mass, ARPES intensity plots along the *X*–*U* and *X*–*Γ* directions are presented in Figure 3c,d, respectively. The electron pockets are fitted with a parabola (red lines). Along *X*–*U*, we obtain *E* − *E*_F_ = (−0.05 ± 0.01) + 5 × *k_y_* ^2^, while along *X*–*Γ*, *E* − *E*_F_ = (−0.05 ± 0.01) + 0.8 × (*k_x_* + 1.06)^2^. According to the formula m* = *ћ* ^2^(*∂* ^2^ *E*/*∂k* ^2^)^−1^, the derived effective mass along *X*–*U* (m_t_*) and *X*–*Γ* (m_l_*) directions is 0.76 and 4.8 *m*_e_, respectively. As a result, the anisotropic factor *K* (*K* = m_l_*/m_t_*) is calculated to be about 6. In comparison, the m_l_* and m_t_* from the calculated electronic structure (Figure 2d) are 0.4 and 3.3 *m*_e_, respectively, giving a *K* value of 8. Combining the results from ARPES and calculations, it is confirmed that the CB of ZrNiSn has a relatively large anisotropic characteristic. Large band anisotropy was also found for the other good thermoelectric materials,^3^, ^4^, ^6^, ^36^ such as PbTe (*K* = 8), SnTe (*K* = 9), and GeTe (*K* = 22). Previously, high band degeneracy *N*_v_ was found contributing to the high performance of half‐Heusler compounds.^20^ Herein, together with the experimentally confirmed large band anisotropy *K*, the so‐called Fermi surface complexity factor *N*_v_ *K* 5 is expected to be high for half‐Heusler compounds, which lays the electronic structure origin of their high electrical PF.

**Figure 3:**
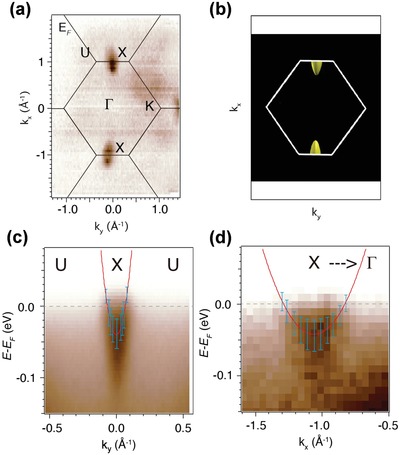
a) Fermi surface intensity map in the *k_x_*–*k_y_* plane measured with the photon energy of 125 eV. b) Fermi surface from first‐principles calculations. ARPES intensity plot along c) *X*–*U* and d) *X*–*Γ*, respectively. The red lines are fitted with a parabola while the blue lines show the error bar set by the ARPES energy resolution.

Moreover, the density of the state effective mass *m*_d_* can be calculated via the expressions: *m*_d_* = *N*_v_^2/3^ *m*_b_* and *m*_b_* = (m_l_* × m_t_*^2^)^1/3^, where *N*_v_ is the band degeneracy and equals to 3 for the CB of ZrNiSn. The derived *m*_d_* from the ARPES data is 2.9 *m*_e_, which shows good consistency with the value of 2.8–3.0 *m*_e_ calculated from the effective mass model^37^ based on the transport data.^19^

To crosscheck the bandgap, we performed optical reflectivity measurement on undoped ZrNiSn single crystal at 10 and 300 K. The derived optical conductivity is presented in Figure S8 in the Supporting Information. To extract the indirect *E*_g‐op_ and direct *E*_g_ from the spectra, we plotted (ε_2_ω^2^)^1/2^ and (ε_2_ω^2^)^2^ versus frequency to estimate the bandgaps, respectively (**Figure** **4**a,b), where ε_2_ is the imaginary part of the complex dielectric function. The extrapolations of the linear parts of these spectra intersect the axis of abscissa providing the values of the bandgaps.^38^ As a result, an indirect *E*_g_op_ of 0.45 ± 0.1 eV was derived, which is in agreement with *E*_g_FP_, but smaller than that of *E*_g_ARPES_. Different experimental techniques generally have different uncertainty of energy resolution, which might explain the deviation of *E*_g_ from the optical and ARPES measurements. Moreover, a direct bandgap of about 1 eV was also derived for ZrNiSn (Figure 4b), corresponding to the minimum direct bandgap of 0.9 eV at *X* point from the calculated electronic structure (Figure 2d).

**Figure 4:**
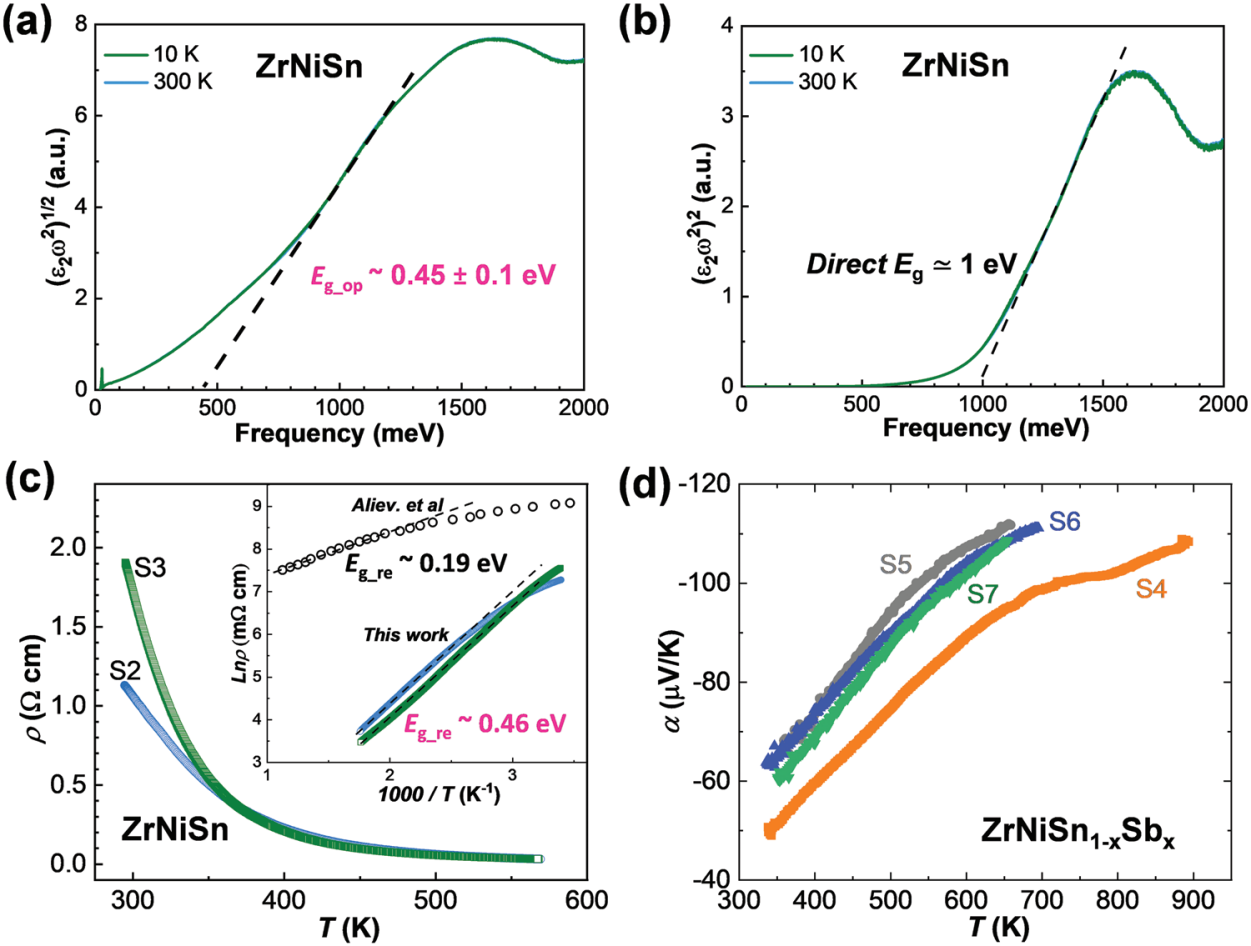
Optical estimation of the indirect a) *E*_g_op_ and the b) direct *E*_g_, respectively. c) Electrical resistivity versus temperature for two undoped ZrNiSn single crystals, denoted as S2 and S3, respectively. The inset shows the *E*_g_re_ estimated using the formula: ρ ≈ exp(*E*_g_re_/2*k*_B_ *T*). The polycrystalline data are taken from Aliev et al.^17^ d) Temperature‐dependent Seebeck coefficient for four Sb‐doped single crystals denoted as S4 to S7.

Furthermore, the electrical resistivity ρ of undoped ZrNiSn single crystals above room temperature was measured and shown in Figure 4c. The ρ exhibits a typical semiconducting behavior. The bandgap *E*_g_re_ is estimated using the high‐temperature resistivity data, as shown in the inset of Figure 4c. *E*_g_re_ of 0.46 eV is obtained for the studied single crystals, which is twice larger than the one obtained from polycrystalline samples by Aliev et al.^17^ The derived bandgap from electrical resistivity shows a reasonable agreement with the ones from first‐principles calculations, and the ARPES and optical measurements. Therefore, it can be concluded that the low‐temperature growth technique used in this work guarantees high‐quality ZrNiSn samples with fewer Ni interstitial defects, enabling the manifestation of the unexpectedly large intrinsic bandgap.

The large bandgap is generally desired for high peak *zT* at elevated temperatures, as it can effectively suppress the thermal excitation of minority carriers and bipolar effect. The comparison of peak *zT* values for the three famous half‐Heusler thermoelectric systems *M*NiSn, *M*CoSb, and *R*FeSb is shown in Figure S9 in the Supporting Information. The *zT* of *M*NiSn system peaks between 800 and 1000 K, whereas the *zT* of *M*CoSb and *R*FeSb does not culminate even above 1100 K. The reason for this difference lies in that *M*NiSn system, synthesized by high‐temperature technique, generally has excess Ni interstitial defects and thus shows a smaller *E*_g_ (0.2–0.3 eV,^21^ Figure 1a). In contrast, the *E*_g_ is generally larger than 0.5 eV for *M*CoSb and *R*FeSb systems.^13^, ^14^ The above results demonstrate that *M*NiSn crystals with fewer Ni interstitial defects could be grown with low‐temperature technique, which enables the manifestation of the unexpectedly large intrinsic *E*_g_ (0.5–0.6 eV) in *M*NiSn. Therefore, a higher peak *zT* might be achieved at higher temperatures in *n*‐type *M*NiSn with fewer Ni interstitial defects to match its *p*‐type counterpart (*M*CoSb and *R*FeSb).

It is thus interesting to further check the Seebeck coefficient of the as‐grown single crystals above room temperature. Due to the small size of single crystals, it is not possible to measure the Seebeck coefficient with commercial ZEM‐3 and Linseis LSR‐3 systems. Herein, a home‐made setup with a two‐probe configuration^39^ was employed to measure the Seebeck coefficient of the single crystals. It is challenging to get reliable Seebeck coefficient of the undoped crystals due to the large contact resistance, but we succeed in measuring the Seebeck coefficient of heavily doped single crystals. As exhibited in Figure 4d, the absolute Seebeck coefficient of these heavily doped crystals shows a degenerate‐semiconductor behavior and keeps increase up to 900 K, which is the upper limit temperature of the home‐made setup. It is expected that the Seebeck coefficient should further rise with increasing temperature above 900 K.

*M*NiSn system with fewer Ni interstitial defects demonstrates a large intrinsic bandgap and thus suppressed bipolar effect. Moreover, higher carrier mobility and lattice thermal conductivity are also expected due to the lack of point defect scattering.^40^ Therefore, distinct from utilizing the Ni interstitial defects to suppress the lattice thermal conductivity, the investigation of the intrinsic electronic structure in this work demonstrates a new direction of half‐Heusler thermoelectric research, namely, enhancing the electrical properties by eliminating the intrinsic defects. Some very recent experimental works on Ni‐poor TiNiSn system have shown that it is indeed promising to improve the electrical power factor and *zT* value by reducing Ni content.^40^, ^41^ Considering that (Zr,Hf)NiSn‐based compounds generally demonstrate higher *zT* than the TiNiSn system even with excess Ni interstitial defects, it is thus optimistically predicted that higher thermoelectric performance might be achieved in (Zr,Hf)NiSn‐based compounds by eliminating the Ni interstitial defects.

In summary, based on the well understanding of processing–structure–property relationships, high‐quality undoped and Sb‐doped ZrNiSn single crystals with fewer Ni interstitial defects have been grown using the low‐temperature technique. High‐resolution ARPES experiment was successfully carried out to reveal the intrinsic electronic structure for the first time. An unexpectedly large intrinsic bandgap of 0.66 ± 0.1 eV was found by ARPES, which is approximately two to three times higher than the values reported in previous polycrystalline samples with considerable Ni interstitial defects. Moreover, the anisotropic characteristic of the conduction band of ZrNiSn was directly observed and the experimental effective mass and anisotropic factor were derived from the ARPES study. These results demonstrate a feasible paradigm to investigate the electronic structure of the 3D solid materials by using ARPES and provide new insights into the intrinsic electronic structure of the half‐Heusler system which could be helpful to further enhance their thermoelectric performance.

## Experimental Section

*Single Crystal Growth and Characterization*: The undoped ZrNiSn and Sb‐doped ZrNiSn_1−_ *_y_*Sb*_y_* single crystals were grown using the Sn Flux method.^42^ The starting powders of Zr, Ni, Sn, and Sb were mixed together in a molar ratio of 1:1+*x*:10:*y* (*x* = −0.1–0.15, *y* = 0–0.04). Next, the mixture was sealed in a dry quartz tube under high vacuum. The tube was heated up to 1100 °C in 15 h and further dwelled for 24 h. For crystal growth, the tube was slowly cooled down to 650 °C at a rate of 2 °C h^−1^. After the growth process, the liquid Sn flux was removed by either decanting or centrifuging. Some additional Sn on the surface of the crystal was further cleaned by etching in dilute hydrochloric acid. The crystals were checked and oriented at room temperature by a Laue X‐ray diffractometer. The phase purity of the crystals was checked using XRD on a Philips X'pert diffractometer with Cu Kα radiation (λ = 1.54184 Å). To study the microstructure and the actual composition, the crystals were first polished and examined using optical microscopy. The scanning electron microscope (SEM) backscattering images were obtained using SEM (JSM7800F, JEOL). Quantitative electron probe microanalysis of the crystals was carried out using an EDX spectroscopy analyzer (Phoenix V 5.29, EDAX) and a WDX spectrometer (Cameca SX 100) using the pure elements as standards. To further confirm the actual composition of ZrNiSn single crystals, ICP‐OES was carried out. Three independent weights of approximately 5 mg (exactly weighed in) sample were digested with an acid mixture of 2.75 mL HCl, 0.5 mL HNO_3_, and 50 µL HF in the microwave system MLS‐Ethos Plus at 155 °C for 15 min. After being cooled to room temperature, each solution was completely transferred into a volumetric flask (50 mL) and filled up with ultrapure water. All three solutions were analyzed using an Agilent 5100 SVDV ICP‐OES. The matrix‐matched standards for the calibration of the spectrometer were prepared from single‐element standards. The Hf content was below the limit of detection (< 100 ppm).

Longitudinal and Hall resistivities were measured by a standard four‐probe method using the Physical Property Measurement System (PPMS, Quantum Design). The accuracy of the resistivity measurement was ± 3%. Hall carrier concentration *n*_H_ was calculated using the equation *n*_H_ = 1/(*eR*_H_), where *e* is the unit charge and *R*_H_ is the Hall coefficient. The carrier mobility *µ*_H_ was calculated using *µ*_H_ = *R*_H_/ρ. The resistivity above room temperature was measured with a homemade setup using the four‐probe method.

*Optical Measurement*: The optical reflectivity measurements were performed on an undoped ZrNiSn single crystal with a lateral dimension of 2 × 2 mm^2^. The crystal had a polished shiny surface with an orientation along 〈111〉. The optical reflectivity *R* as a function of frequency ω was measured using a standard method^38^ with a Bruker Hyperion microscope attached to a Bruker Vertex 80v Fourier transform spectrometer. Freshly evaporated gold mirrors served as the references. Complex optical conductivity was obtained from *R*(ω) using Kramers–Kronig transformations.^38^ High‐frequency extrapolations were made utilizing the X‐ray atomic scattering functions. For the extrapolations toward zero frequency, a constant (dielectric) reflectivity was assumed.

*ARPES*: The ARPES experiments were conducted at the SIS endstation at Swiss Light Source, with a Scienta R4000 analyzer. The photon energy was in the UV region (20–200 eV). The samples with a typical size of about 1 × 1 × 2 mm^3^ were cleaved at 15 K in high vacuum chamber, with base vacuum higher than 5 × 10^−11^ Torr.

*First‐Principles Calculations*: First‐principles calculations on ZrNiSn were performed using the projector augmented wave method, as implemented in the Vienna ab initio simulation package (VASP).^43^ The Perdew–Burke–Ernzerhof generalized gradient approximation^44^ for the exchange–correlation potential was used for the band structure calculation. The *k*‐mesh of the calculation for the Fermi surface, which was visualized in the XcrySDen package,^45^ was 45 × 45 × 45 for the primitive cell. A plane‐wave energy cutoff of 520 eV and an energy convergence criterion of 10^−4^ eV for self‐consistency were adopted. All the atomic positions were relaxed to equilibrium until the calculated Hellmann–Feynman force on each atom was less than 10^−2^ eV Å^−1^.

## Conflict of Interest

The authors declare no conflict of interest.

## Supporting information

Supporting Information
