# Supplementary material for: Revealing the Intrinsic Electronic Structure of 3D Half‐Heusler Thermoelectric Materials by Angle‐Resolved Photoemission Spectroscopy
Source: Adv Sci (Weinh). 2019 Nov 6;7(1):1902409. doi: 10.1002/advs.201902409 (PMC6947594; doi:10.1002/advs.201902409)
Supplement: Supplementary file 1 — Supporting Information [file ADVS-7-1902409-s001.pdf]

Copyright WILEY-VCH Verlag GmbH & Co. KGaA, 69469 Weinheim, Germany, 2019.

ADVANCED SCIENCE Open Access

# Supporting Information

for *Adv. Sci.,* DOI: 10.1002/advs.201902409

# Revealing the Intrinsic Electronic Structure of 3D Half-Heusler Thermoelectric Materials by Angle-Resolved Photoemission Spectroscopy

*Chenguang Fu,* Mengyu Yao,* Xi Chen, Lucky Zaehir Maulana, Xin Li, Jiong Yang, Kazuki Imasato, Fengfeng Zhu, Guowei Li, Gudrun Auffermann, Ulrich Burkhardt, Walter Schnelle, Jianshi Zhou, Tiejun Zhu, Xinbing Zhao, Ming Shi, Martin Dressel, Artem V. Pronin, G. Jeffrey Snyder, and Claudia Felser**

Copyright WILEY-VCH Verlag GmbH & Co. KGaA, 69469 Weinheim, Germany, 2019.

# Supporting Information

**Revealing the intrinsic electronic structure of 3-dimensional half-Heusler thermoelectric materials by angle-resolved photoemission spectroscopy**

*Chenguang Fu,* Mengyu Yao,* Xi Chen, Lucky Zaehir Maulana, Xin Li, Jiong Yang, Kazuki Imasato, Fengfeng Zhu, Guowei Li, Gudrun Auffermann, Ulrich Burkhardt, Walter Schnelle, Jianshi Zhou, Tiejun Zhu, Xinbing Zhao, Ming Shi, Martin Dressel, Artem V. Pronin, G. Jeffrey Snyder, Claudia Felser**

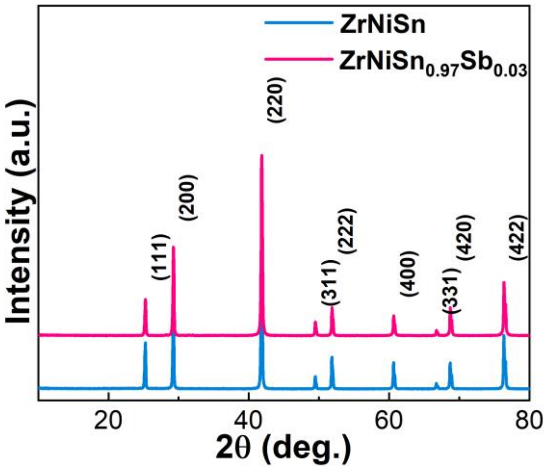


**Figure S1.** Power XRD for undoped ZrNiSn and Sb-doped $ZrNiSn_{0.97}Sb_{0.03}$ crystals.

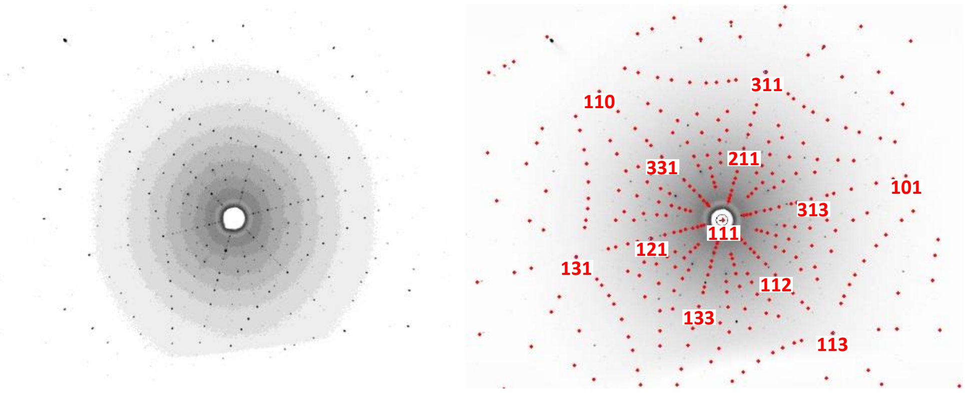


**Figure S2.** Laue diffraction for typical ZrNiSn single crystal.

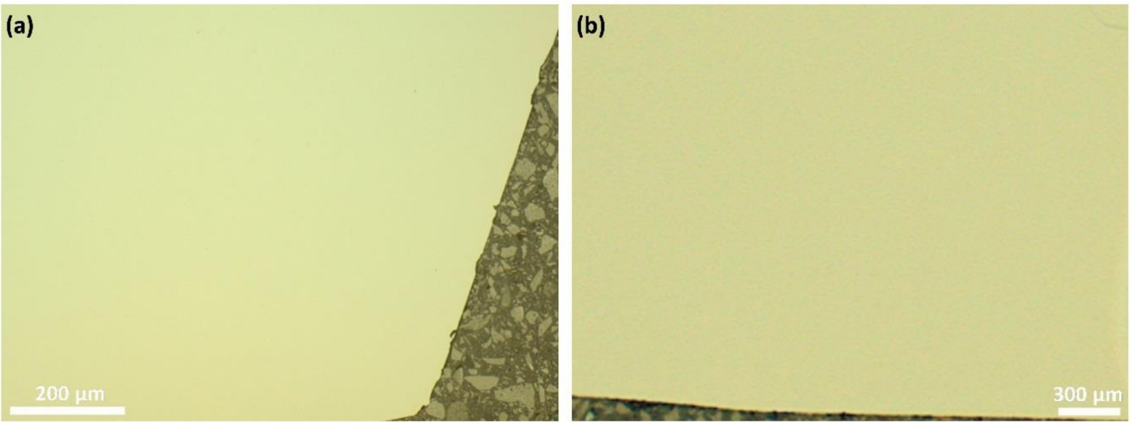


**Figure S3**. Optical microscopy images show the homogeneous phase of ZrNiSn (a) and $ZrNiSn_{0.97}Sb_{0.03}$ (b) single crystals.

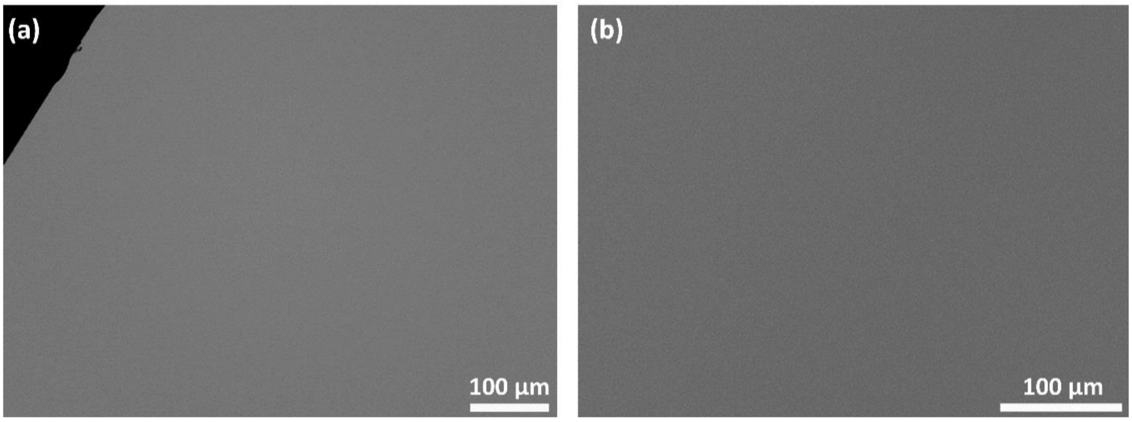


**Figure S4**. The SEM backscattering images for ZrNiSn (a) and $ZrNiSn_{0.97}Sb_{0.03}$ (b) single crystals.

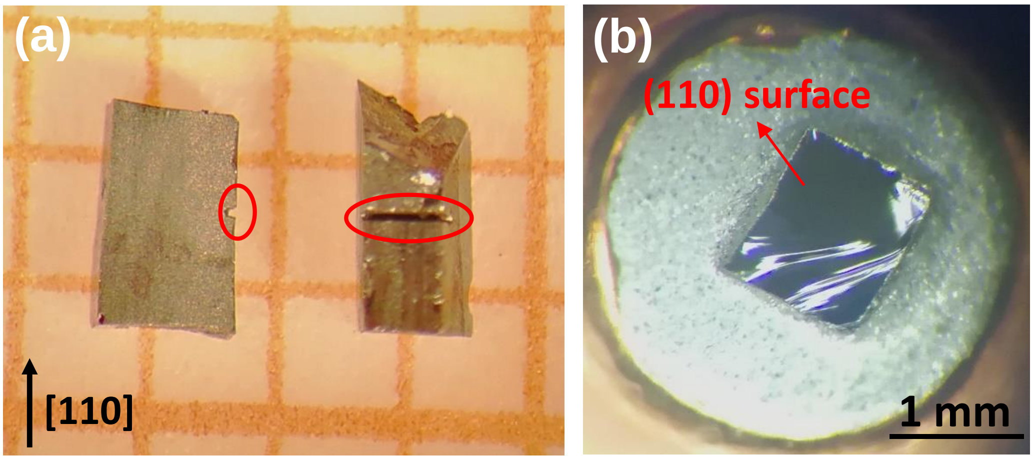


**Figure S5**. (a) Optical image of the oriented single crystals for ARPES study. The red circles highlight the incision made by a 50 μm wire saw. (b) A shiny and flat crystal surface obtained after the cleavage.

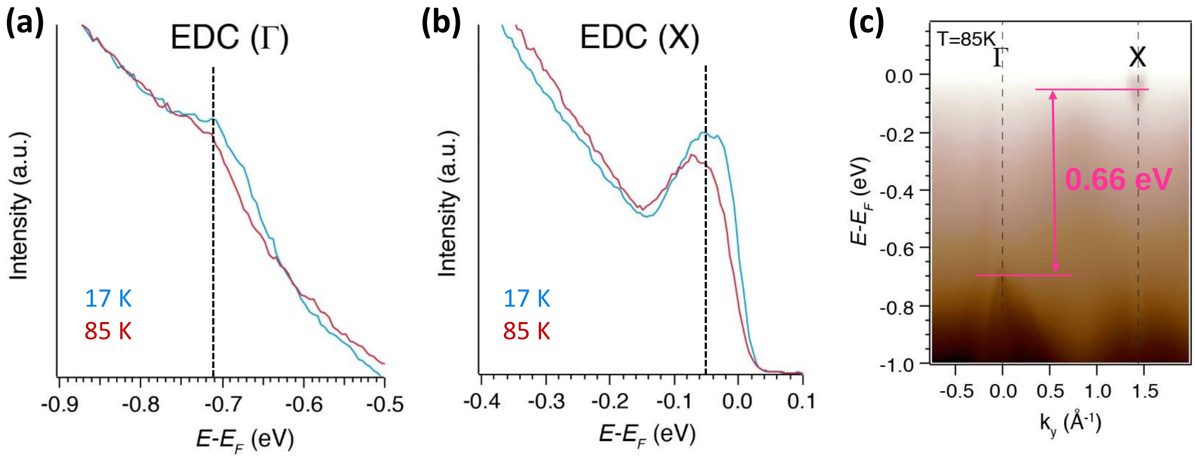


**Figure S6.** The energy distribution curves (EDC) taken at Γ point (a) and X point (b) under 17K (blue line) and 85 K (red line), respectively. (c) ARPES intensity plots along Γ-X, taken with the photon energy of 125 eV at 85 K.

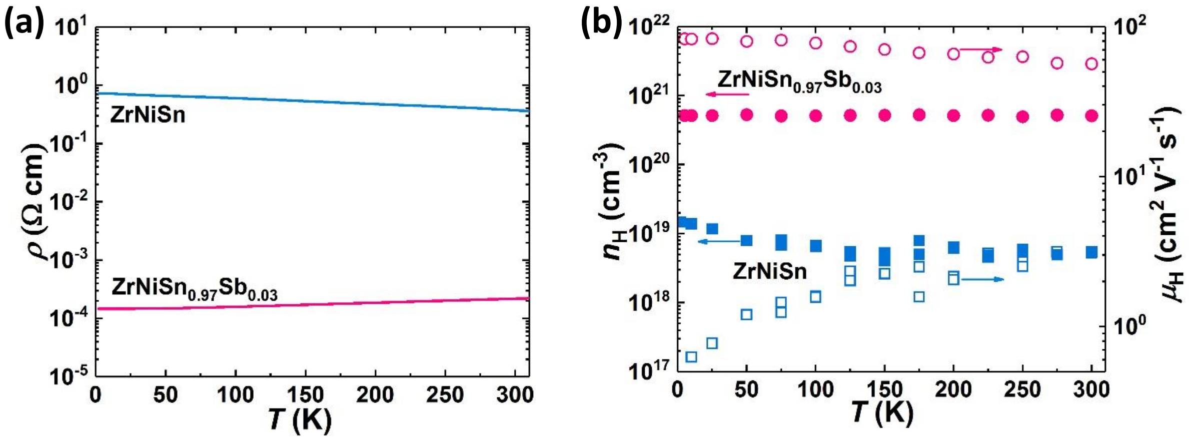


**Figure S7.** Temperature-dependent resistivity (a), Hall carrier concentration and mobility (b) for the undoped ZrNiSn and Sb-doped $ZrNiSn_{0.97}Sb_{0.03}$ single crystals.

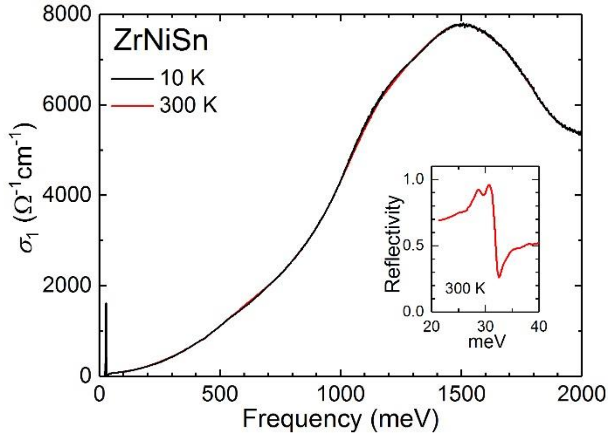


**Figure S8**. The real part of the complex optical conductivity ($\sigma_1$) of the undoped ZrNiSn single crystal. The inset shows the optical reflectivity at low energies.

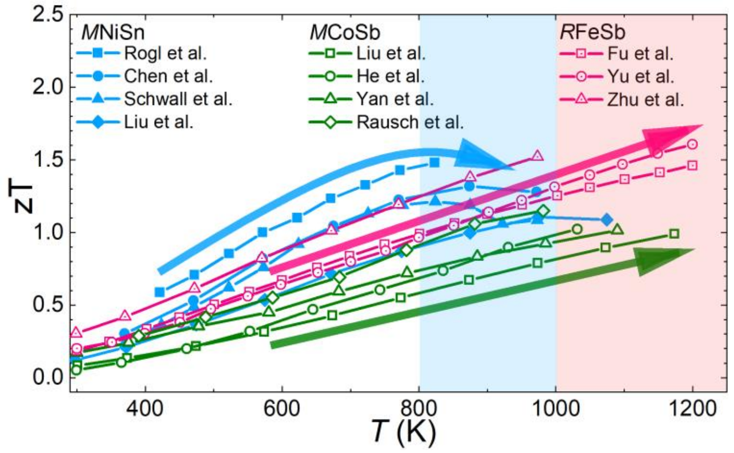


**Figure S9**. *zT* comparison for the most famous three half-Heusler thermoelectric systems: *M*NiSn[S1], *M*CoSb[S2], and *R*FeSb [S3]. The arrows show the trends of the *zT* values.

**Table S1**. Crystallographic data for ZrNiSn from powder XRD refinement.

| | | | | | |
|---|---|---|---|---|---|
| a (Å): | 6.1033(2) | | Two-theta (max) | | 100.30 |
| Cell volume (A$^3$) | 227.35(3) | | sinT/l (max) | | 0.498 |
| F(000) (electrons) | 472.0 | | h(min), k(min), l(min) | | 0 0 1 |
| Number of atoms in cell | 12.0 | | h(max), k(max), l(max) | | 3 4 6 |
| Calculated density (g/cm$^3$) | 7.8473(9) | | R(I), R(p) | | 0.0293, 0.1424 |
| Absorption coefficient (1/cm) | 1347.09 | | Goodness of fit | | 3.980 |
| Radiation and wavelength | Cu K(α1) 1.54056 | | Scale factor | | 0.06689(0) |
| Diffractometer | Powder | | Number of free parameters | | 4 |
| Mode of refinement | Full profile | | Number of atom sites | | 3 |
| | | | | | |
| | All | Sc | Atv. | Pv | B | Atoms |
| All | 23 | 2 | 21 | 12 | 9 | 3 All |
| Free | 4 | 1 | 3 | 0 | 3 | 3 Iso |
| Fixed | 19 | 1 | 18 | 12 | 6 | 0 Aniso |
| | | | | | | |
| Contents: | Atom | No. in cell | Atomic % | Weight % | | |
| | Zr | 4 | 33.333 | 33.959 | | |
| | Ni | 4 | 33.333 | 21.856 | | |
| | Sn | 4 | 33.333 | 44.185 | | |
| | | | | | | |
| Atomic parameters for ZrNiSn | | | | | | |
| Atom | x/a | y/b | z.c | B(is/eq) | N | |
| Zr1 | 0 | 0 | 0 | 1.4 (2) | 4 | |
| Sn1 | 1/2 | 1/2 | 1/2 | 0.75 (11) | 4 | |
| Ni1 | 1/4 | 1/4 | 1/4 | 1.3 (3) | 4 | |
| | | | | | | |
| Berar’s factor | 7.12 (apply). | | | | | |

**Table S2**. EDX analysis for undoped ZrNiSn and Sb-doped $ZrNiSn_{0.97}Sb_{0.03}$ single crystals performed on 6 randomly selected positions. The average atomic percentage is shown in the last row.

| **EDX** | **Undoped ZrNiSn** | | | **EDX** | **Sb-doped $ZrNiSn_{0.97}Sb_{0.03}$** | | | |
|---|---|---|---|---|---|---|---|---|
| position | Zr (%) | Ni (%) | Sn (%) | position | Zr (%) | Ni (%) | Sn (%) | Sb (%) |
| 1 | 33.28 | 33.62 | 33.09 | 1 | 33.42 | 33.9 | 31.56 | 1.12 |
| 2 | 33.41 | 33.83 | 32.75 | 2 | 33.87 | 33.63 | 31.37 | 1.12 |
| 3 | 33.19 | 33.79 | 33.02 | 3 | 32.93 | 34.1 | 32.02 | 0.95 |
| 4 | 33.54 | 34.14 | 32.32 | 4 | 33.26 | 33.83 | 31.66 | 1.24 |
| 5 | 33.11 | 33.73 | 33.16 | 5 | 33.25 | 34.39 | 31.12 | 1.23 |
| 6 | 33.56 | 34.01 | 32.42 | 6 | 33.48 | 33.75 | 31.47 | 1.31 |
| *average* | *33.35* | *33.85* | *32.79* | *average* | *33.37* | *33.93* | *31.53* | *1.16* |

**Table S3**. WDX analysis for undoped ZrNiSn and Sb-doped $ZrNiSn_{0.97}Sb_{0.03}$ single crystals performed on 10 randomly selected positions. The average weight % with standard deviation in the parentheses for each element is summarized in the last row.

| **WDX** | **Undoped ZrNiSn** | | | **Sb-doped $ZrNiSn_{0.97}Sb_{0.03}$** | | | |
|---|---|---|---|---|---|---|---|
| position | Zr / Weight % | Ni / Weight % | Sn / Weight % | Zr / Weight % | Ni / Weight % | Sn / Weight % | Sb / Weight % |
| 1 | 33.37 | 22.35 | 44.61 | 34.22 | 22.50 | 43.23 | 1.29 |
| 2 | 33.50 | 22.45 | 44.55 | 34.13 | 22.45 | 43.13 | 1.16 |
| 3 | 33.42 | 22.47 | 44.59 | 34.40 | 22.40 | 43.17 | 1.33 |
| 4 | 33.46 | 22.48 | 44.51 | 34.29 | 22.40 | 43.11 | 1.50 |
| 5 | 33.42 | 22.40 | 44.52 | 34.28 | 22.45 | 43.01 | 1.44 |
| 6 | 33.51 | 22.50 | 44.60 | 34.31 | 22.45 | 43.06 | 1.33 |
| 7 | 33.57 | 22.52 | 44.58 | 34.24 | 22.43 | 43.16 | 1.28 |
| 8 | 33.50 | 22.48 | 44.63 | 34.30 | 22.47 | 42.94 | 1.69 |
| 9 | 33.27 | 22.43 | 44.57 | 34.15 | 22.37 | 43.00 | 1.29 |
| 10 | 33.37 | 22.43 | 44.49 | 34.19 | 22.45 | 43.03 | 1.43 |
| **average** | 33.44 (0.11) | 22.45 (0.08) | 44.56 (0.12) | 34.25 (0.11) | 22.44 (0.08) | 43.08 (0.11) | 1.38 (0.01) |

**Table S4.** ICP analysis for the undoped ZrNiSn single crystal.

| **ICP** | **ZrNiSn** | | |
|---|---|---|---|
| | Zr / Weight % | Ni / Weight % | Sn / Weight % |
| **average** | 33.93 (0.47) | 22.15 (0.31) | 44.22 (0.61) |

## References

[S1] M. Schwall, B. Balke, *Phys. Chem. Chem. Phys.* **2013**, *15*, 1868; Y. Liu, H. Xie, C. Fu, G. J. Snyder, X. Zhao, T. Zhu, *J. Mater. Chem. A* **2015**, *3*, 22716; L. Chen, X. Zeng, T. M. Tritt, S. J. Poon, *J. Electron. Mater.* **2016**, *45*, 5554; G. Rogl, P. Sauerschnig, Z. Rykavets, V. V. Romaka, P. Heinrich, B. Hinterleitner, A. Grytsiv, E. Bauer, P. Rogl, *Acta Mater*. **2017**, *131*, 336.

[S2] X. Yan, W. Liu, H. Wang, S. Chen, J. Shiomi, K. Esfarjani, H. Wang, D. Wang, G. Chen, Z. Ren, *Energy Environ. Sci.* **2012**, *5*, 7543; Y. Liu, C. Fu, K. Xia, J. Yu, X. Zhao, H. Pan, C. Felser, T. Zhu, *Adv. Mater.* **2018**, *30*, 1800881; R. He, H. Zhu, J. Sun, J. Mao, H. Reith, S. Chen, G. Schierning, K. Nielsch, Z. Ren, *Mater. Today Phys*. **2017**, *1*, 24.

[S3] C. Fu, S. Bai, Y. Liu, Y. Tang, L. Chen, X. Zhao, T. Zhu, *Nat. Commun.* **2015**, *6*, 8144; J. Yu, C. Fu, Y. Liu, K. Xia, U. Aydemir, T. C. Chasapis, G. J. Snyder, X. Zhao, T. Zhu, *Adv. Energy Mater*. **2018**, *8*, 1701313; H. Zhu, J. Mao, Y. Li, J. Sun, Y. Wang, Q. Zhu, G. Li, Q. Song, J. Zhou, Y. Fu, R. He, T. Tong, Z. Liu, W. Ren, L. You, Z. Wang, J. Luo, A. Sotnikov, J. Bao, K. Nielsch, G. Chen, D. J. Singh, Z. Ren, *Nat. Commun*. **2019**, *10*, 270.
